# Green-Synthesized Silver Nanoparticles from Garlic Peel Target NF-κB and Redox Imbalance: A Novel Therapeutic Strategy Against Pyrogallol-Induced Hepatotoxicity in Rats

**DOI:** 10.3390/nano15211610

**Published:** 2025-10-22

**Authors:** Duaa A. Althumairy

**Affiliations:** Department of Biological Sciences, College of Science, King Faisal University, P.O. Box 380, Al-Ahsa 31982, Saudi Arabia; dalthumairy@kfu.edu.sa; Tel.: +966-505915104

**Keywords:** green synthesis, garlic peel extract, silver nanoparticles, hepatoprotection, oxidative stress, NF-κB signaling, sustainable nanomedicine

## Abstract

Background/Objectives: Hepatotoxicity remains a major therapeutic challenge driven by oxidative stress and inflammation. This study investigated the hepatoprotective potential of green-synthesized silver nanoparticles derived from ethanolic garlic peel extract (GPE-Ag) against pyrogallol-induced liver injury. Methods: Adult rats were randomly assigned into four groups: a control group, a pyrogallol-treated group, a group receiving GPE-Ag nanoparticles (50 mg/kg, orally) for 28 days, and GPE-Ag + pyrogallol co-treated. Results: The garlic peel extract was analyzed by high-performance liquid chromatography (HPLC), revealing high levels of phenolic acids (66.83 µg/g) and flavonoids (59.81 µg/g), predominantly ellagic, gallic, and syringic acids, along with kaempferol, quercetin, and myricetin. The synthesized GPE-Ag nanoparticles were characterized using UV–Vis spectroscopy, transmission and scanning electron microscopy (TEM and SEM), zeta potential, dynamic light scattering (DLS), and energy-dispersive X-ray analysis (EDAX). GPE-Ag treatment markedly attenuated pyrogallol-induced hepatic injury by reducing serum liver enzyme levels, lipid peroxidation, and proinflammatory cytokines, including interleukin-1 (IL-1), interleukin-6 (IL-6), tumor necrosis factor-alpha (TNF-α), and nuclear factor-kappa B (NF-κB), while enhancing the activities of antioxidant enzymes, catalase (CAT) and glutathione peroxidase (GPx), as well as the anti-inflammatory cytokine interleukin-10 (IL-10). Histological examination further confirmed the restoration of normal hepatic architecture. Conclusion: This study provides the first evidence that garlic peel–derived silver nanoparticles exert potent hepatoprotective effects through redox homeostasis restoration and modulation of the NF-κB signaling pathway. These findings highlight GPE-Ag as a promising, sustainable nanotherapeutic candidate for managing chemically induced liver injury.

## 1. Introduction

Hepatotoxicity, a pathological condition frequently triggered by xenobiotics, environmental pollutants, and oxidative stress, remains a significant contributor to global morbidity and mortality [1,2,3]. As the principal organ responsible for metabolic processing and detoxification, the liver is especially vulnerable to chemical and drug-induced insults. Hepatic injury is often characterized by oxidative damage, inflammatory responses, and hepatocellular apoptosis, which compromise liver function and can lead to chronic liver diseases, including fibrosis and cirrhosis [4,5,6]. One of the primary signaling mechanisms involved in liver inflammation is NF-κB pathway, which regulates the transcription of various pro-inflammatory cytokines, chemokines, and adhesion molecules, thereby promoting tissue damage and the progression of hepatic disorders [7,8,9,10].

Pyrogallol (1,2,3-trihydroxybenzene), a naturally occurring phenolic compound found in many plants, is widely used to induce experimental hepatotoxicity due to its potent pro-oxidant properties and ability to trigger hepatic inflammation [11]. It significantly increases the expression of inflammatory cytokines and upregulates Cyclooxygenase-2 (COX-2) and NF-κB signaling in vivo [12,13]. Pyrogallol’s pathological effects include inducing lipid peroxidation, disrupting mitochondrial function, causing DNA fragmentation, and weakening the endogenous antioxidant defense system. These properties make it a valuable model for assessing potential hepatoprotective interventions [14,15,16].

Over recent years, increasing interest has been directed toward plant-based compounds and herbal extracts as alternatives or complements to traditional hepatoprotective agents. Standard pharmacological treatments such as N-acetylcysteine (NAC), ursodeoxycholic acid (UDCA), S-adenosylmethionine (SAMe), and L-ornithine L-aspartate (LOLA) offer hepatoprotection by targeting oxidative stress, glutathione metabolism, bile acid regulation, and ammonia clearance [17,18,19,20]. Likewise, natural substances like silymarin and curcumin have been shown to exert antioxidant, anti-inflammatory, and antifibrotic properties [21]. However, synthetic drugs may cause long-term side effects, and many natural compounds face challenges such as low solubility, poor bioavailability, and inconsistent pharmacokinetics [22,23].

Nanotechnology, particularly the green synthesis of silver nanoparticles (AgNPs), has emerged as a promising approach that combines the advantages of natural and synthetic methods. This eco-friendly process utilizes plant-derived phytochemicals as reducing and stabilizing agents, eliminating toxic reagents and enhancing the biological activity, solubility, and surface interactions of the resulting nanoparticles, thereby improving their clinical potential [24,25,26,27].

Silver nanoparticles, in particular, are attractive due to their biocompatibility, large surface area, and diverse pharmacological properties, including antioxidant, anti-inflammatory, and antimicrobial activities relevant to liver injury [28,29,30,31]. Previous studies suggest that green-synthesized AgNPs are effective in reducing oxidative stress and hepatic inflammation [32,33,34].

The growing prevalence of liver disorders underscores the need for safer and more effective therapeutic approaches, particularly those that can address chemically induced hepatocellular damage [35,36,37,38]. Silver nanoparticles exhibit strong antioxidant and hepatoprotective effects, mainly due to phenolic compounds such as catechol, caffeic acid, and pyrogallol, which act as natural reducing and stabilizing agents. These phenolics convert silver ions (Ag^+^) into metallic silver (Ag^0^), thereby enhancing the biological activity and therapeutic potential of the nanoparticles [27,39,40,41]. Recent mechanistic studies have demonstrated that plant-derived phenolics activate the Nrf2/HO-1 and AMPK–SIRT1 pathways while inhibiting NF-κB activation, thereby enhancing antioxidant defenses and reducing cytokine-mediated liver injury. These mechanisms likely underlie the synergistic hepatoprotective effects of GPE-Ag treatment [42,43,44].

Garlic peel, an agro-industrial byproduct, is an excellent source of phytochemicals suited for green nanoparticle synthesis. Compounds in garlic peel have been shown to reduce oxidative damage and inflammation in hepatic models [45,46,47]. Our prior findings, along with other reports, confirm that garlic peel extract provides strong antioxidant, anti-apoptotic, and anti-inflammatory activities due to its rich content of bioactive molecules [48,49]. Similar beneficial effects have been observed in nanoparticles synthesized from garlic and other vegetable peels, validating their therapeutic relevance [50,51,52,53,54].

In the current study, GPE-Ag was shown to inhibit NF-κB signaling, regulate inflammatory cytokines, and exhibit strong antioxidant activity, which may be attributed to its high content of phenolic acids and flavonoids, highlighting its multifunctional potential for liver protection.

Despite previous reports on silver nanoparticles, the bioactivity of garlic peel–mediated AgNPs (GPE-Ag) in liver injury models remains underexplored. This study synthesized eco-friendly GPE-Ag using polyphenol-rich garlic peel waste and evaluated their hepatoprotective potential via antioxidant, cytokine, and NF-κB assessments. HPLC analysis identified the main bioactive components responsible for nanoparticle formation and activity. Together with the pyrogallol-induced hepatotoxicity model, these findings establish GPE-Ag as a novel, sustainable nanoplatform with potent antioxidant and anti-inflammatory effects.

## 2. Results

### 2.1. Identification of Polyphenols Profile of Garlic Peel Extract (GPE) Determined by HPLC Analysis

**Figure 1:**
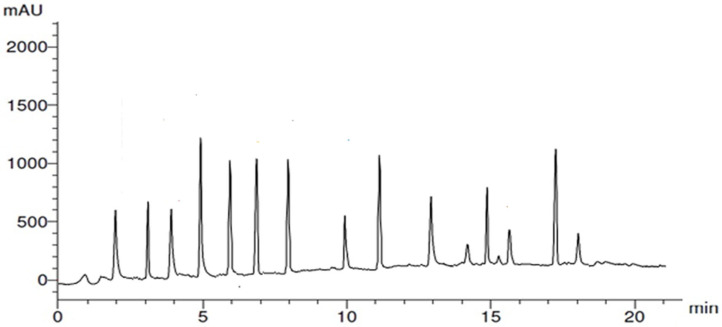
HPLC chromatogram of garlic peel extract (GPE).

The HPLC analysis of garlic peel extract (GPE), summarized in Table 1 and Figure 1, revealed a diverse array of polyphenolic compounds. The major constituents, based on concentration (µg/g), were ellagic acid (18.78 µg/g), gallic acid (15.20 µg/g), kaempferol (15.67 µg/g), quercetin (14.77 µg/g), syringic acid (13.89 µg/g), and myricetin (13.76 µg/g). Other identified compounds included chlorogenic acid (6.20 µg/g), ferulic acid (7.94 µg/g), benzoic acid (7.64 µg/g), 7-hydroxyflavone (4.88 µg/g), apigenin (4.63 µg/g), rutin (4.25 µg/g), catechin (1.85 µg/g), cinnamic acid (2.04 µg/g), and salicylic acid (3.08 µg/g). These polyphenols contributed to the reduction of Ag^+^ ions and stabilization of GPE-Ag nanoparticles. The results indicate that garlic peel is a rich source of polyphenols with potent reducing and capping capabilities.

### 2.2. UV–Vis Spectroscopic Analysis of GPE-Ag Nanoparticles

**Figure 2:**
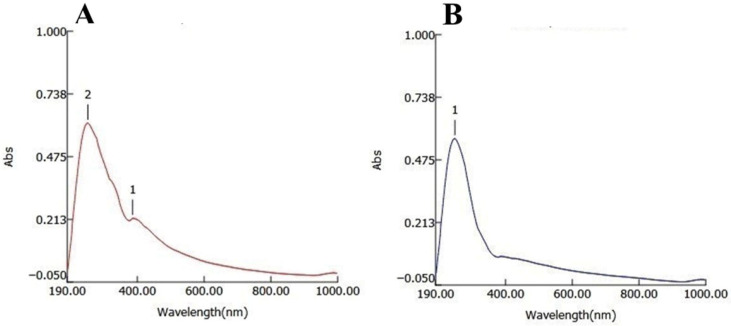
UV–visible spectra of synthesized GPE-Ag (**A**) and the control sample (**B**).

UV–visible spectroscopy analysis confirmed the successful synthesis of silver nanoparticles using ethanolic garlic peel extract (GPE-Ag). A prominent absorption peak appeared at 386 nm (Figure 2A), which falls within the typical surface plasmon resonance (SPR) range (380–420 nm) for silver nanoparticles. This peak is associated with the localized surface plasmon resonance (LSPR) phenomenon, which depends on the nanoparticles’ size, shape, and distribution. In contrast, the control sample (Figure 2B) exhibited no absorbance in the SPR region, indicating the absence of silver nanoparticle formation. The presence of phytochemicals in the garlic peel extract likely played a dual role in reducing silver ions and stabilizing the synthesized nanoparticles.

### 2.3. TEM, SEM and Size Analysis of GPE-Ag Nanoparticles

**Figure 3:**
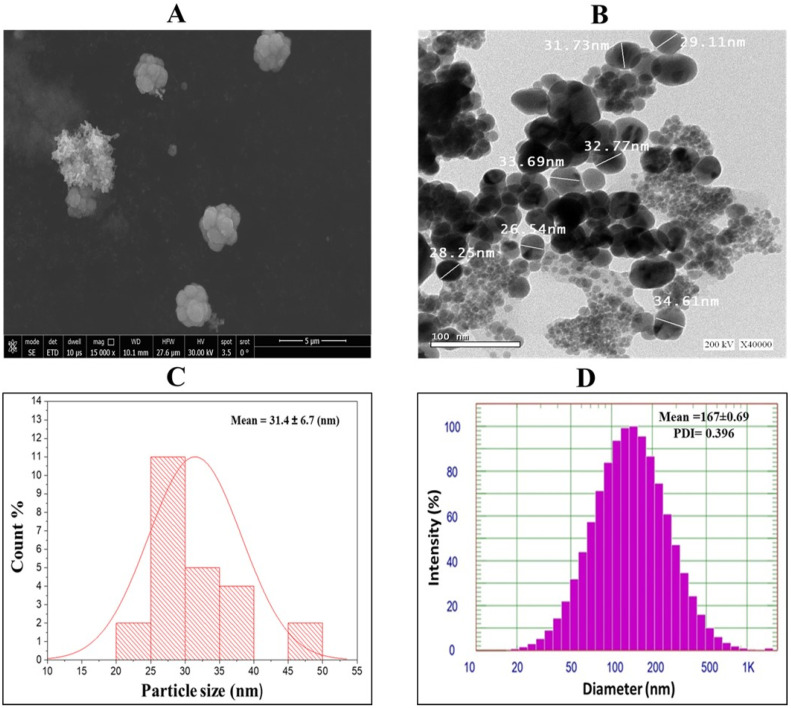
(**A**) Scanning electron microscopy (SEM) image, (**B**) TEM image, (**C**) particle size distribution analyzed from TEM images using ImageJ software, and (**D**) dynamic light scattering (DLS) analysis showing particle size and polydispersity index (PDI).

SEM analysis revealed the uniform morphology and surface topology of the synthesized silver nanoparticles (GPE-Ag) (Figure 3A). TEM analysis further showed that the nanoparticles were predominantly spherical, moderately dispersed, and exhibited minimal agglomeration. TEM was performed using a JEM-2100 electron microscope (JEOL Ltd., 3-1-2 Musashino, Akishima, Tokyo 196-8558, Japan) operated at 200 kV to examine the morphology and particle size of the synthesized nanoparticles (Figure 3B). The TEM images were analyzed using ImageJ software (version 1.53k; National Institutes of Health, Bethesda, MD, USA), revealing a metallic core size ranging from 20 to 40 nm, with an average diameter of 31.4 ± 6.7 nm (SD) (Figure 3C). DLS analysis of the nanoparticle suspension revealed a larger hydrodynamic diameter of approximately 167 ± 0.63 nm (SD) with a polydispersity index (PDI) of 0.396, indicating a moderately polydisperse nanoparticle population. This size distribution likely reflects the influence of the hydration layer and polyphenolic capping on the nanoparticle surface (Figure 3D).

### 2.4. Elemental Composition Analysis of GPE-Ag Nanoparticles by Energy-Dispersive X-Ray (EDX) Spectroscopy

**Figure 4:**
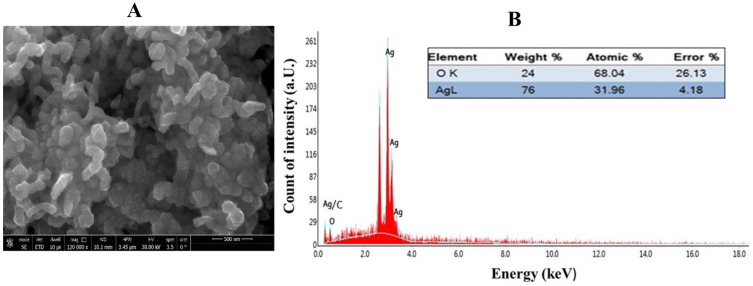
(**A**) EDAX- SEM Map and (**B**) its spectrum of GPE-Ag nanoparticles. The experımental and fıtted data are overlaid (keV) stands for kilo-electronvolt, (a.u.) Arbitrary Unit. O K (Oxygen detected via Kα emission) and AgL (Silver detected via Lα emission).

The elemental composition of the biosynthesized silver nanoparticles (GPE-Ag) was evaluated using EDAX integrated with SEM. EDAX elemental mapping of the selected area (Area 1, Figure 4A) and the corresponding spectrum (Figure 4B) confirmed the presence of silver (Ag) and oxygen (O) as the main constituents. Quantitative analysis showed that silver constituted 76% by weight, while oxygen contributed 24% by weight. A strong characteristic peak for silver was observed at approximately 3 keV, corresponding to the characteristic X-ray emission of elemental silver, which confirms its successful reduction and incorporation into the nanoparticle structure. The presence of oxygen further supports the involvement of plant-derived biomolecules as natural capping and stabilizing agents. Notably, the absence of any additional elemental peaks indicates the high purity of the synthesized GPE-Ag.

### 2.5. Zeta Potential Analysis of GPE-Ag Nanoparticles

**Figure 5:**
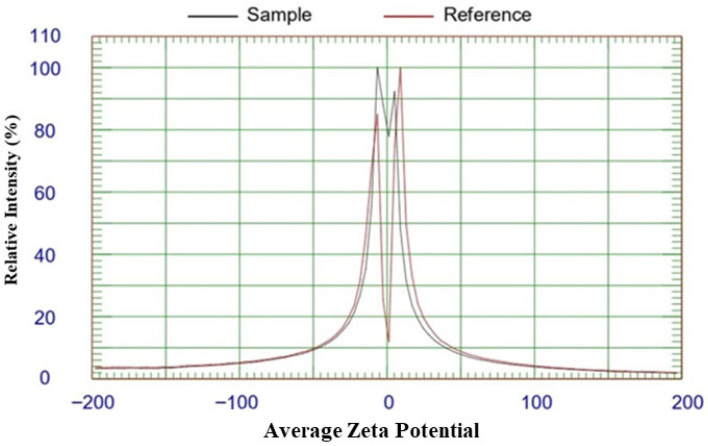
Average zeta potential distribution of GPE-Ag nanoparticles.

The zeta potential measurement of biosynthesized silver nanoparticles (GPE-Ag) using a ZetaPALS analyzer (Figure 5) revealed an average zeta potential (−26.92 mV; −0.80 Hz; average mobility −1.88 M.U; 25 °C; PH, 7.4). This negative surface charge originates from deprotonated hydroxyl (–O^−^) and carboxyl (–COO^−^) groups of polyphenolic compounds present in the garlic peel extract. The strong negative potential confirms effective capping, reduction, and good colloidal stability of the biosynthesized nanoparticles.

### 2.6. Effect of GPE-Ag Nanoparticles on Liver Function Enzymes in Pyrogallol-Intoxicated Rats

**Figure 6:**
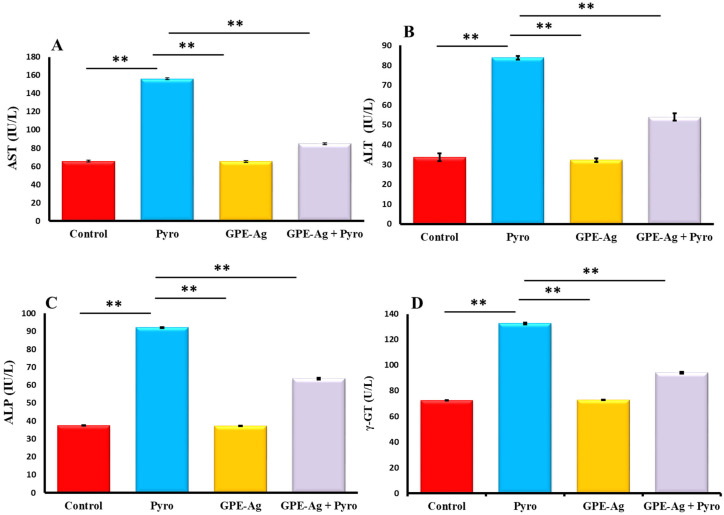
Effect of garlic peel extract-synthesized silver nanoparticles (GPE-Ag) on serum hepatic function enzymes in pyrogallol (Pyro)-intoxicated rats. Serum levels of (**A**) AST, (**B**) ALT, (**C**) ALP, and (**D**) GGT were measured across the experimental groups. Data expressed as mean values ± SE (*n* = 6/group). ** indicates *p* < 0.001 vs. the Pyro group.

As presented in Figure 6A–D, serum activities of aspartate aminotransferase (AST) (Figure 6A), alanine aminotransferase (ALT) (Figure 6B), alkaline phosphatase (ALP) (Figure 6C), and gamma-glutamyl transferase (GGT) (Figure 6D) were significantly increased in the pyrogallol (Pyro) group compared to the control group (*p* < 0.001), indicating liver injury. In contrast, the garlic peel-extract-synthesized silver nanoparticle (GPE-Ag) group showed no significant changes compared to the control group (*p* > 0.05). Co-treatment with GPE-Ag and pyrogallol (GPE-Ag + Pyro) resulted in a significant elevation of these enzyme levels compared to control (*p* < 0.001); however, the increases were markedly lower than those observed in the Pyro group. Both the GPE-Ag and GPE-Ag + Pyro groups showed significant reductions in all enzyme activities relative to the Pyro group (*p* < 0.001), reflecting the protective role of GPE-Ag against pyrogallol-induced hepatotoxicity.

### 2.7. Effect of GPE-Ag Nanoparticles on Hepatic Inflammatory Markers in Pyrogallol-Intoxicated Rats

**Figure 7:**
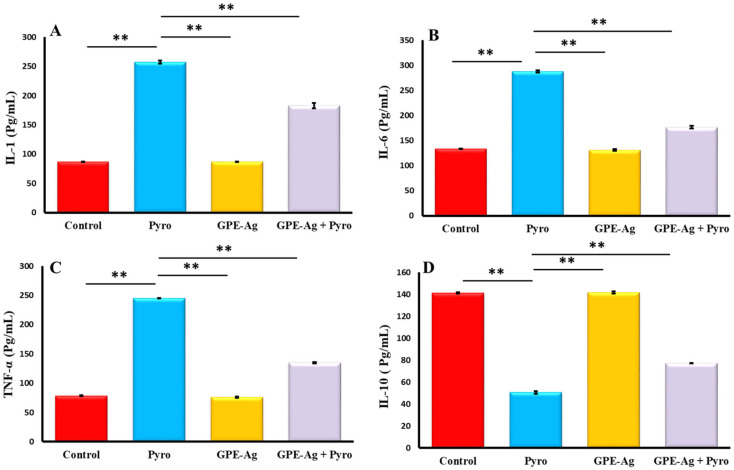
Effect of garlic peel extract-synthesized silver nanoparticles (GPE-Ag) on hepatic pro- and anti-inflammatory mediators in pyrogallol (Pyro)-intoxicated rats. (**A**) IL-1, (**B**) IL-6, (**C**) TNF-α, (**D**) IL-10 detected by ELLISA kits Data expressed as mean values ± SE (*n* = 6/group). ** indicates *p* < 0.001 vs. the Pyro group.

**Figure 8:**
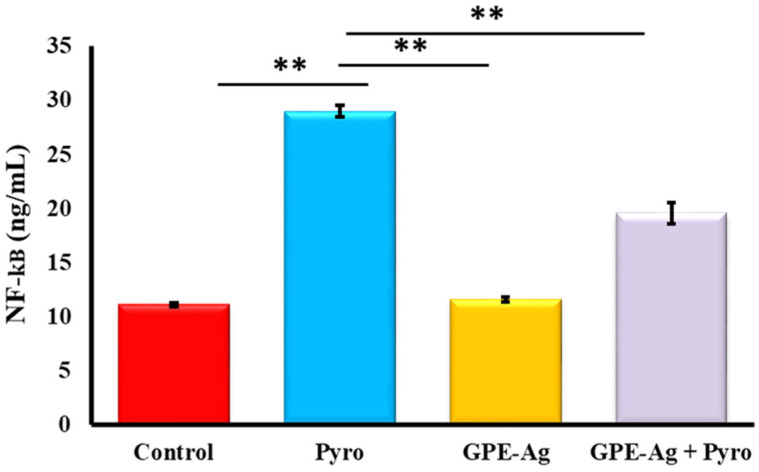
Effect of garlic peel extract-synthesized silver nanoparticles (GPE-Ag) on hepatic NF-κB activity in pyrogallol (Pyro)-intoxicated rats. NF-κB levels were directly quantified in liver tissue homogenates using a rat-specific sandwich enzyme immunoassay kit. Data are presented as mean ± SE (*n* = 6 per group). ** *p* < 0.001 vs. the Pyro group.

As shown in Figure 7A–C, and Figure 7 the hepatic levels of IL-1 (Figure 7A), IL-6 (Figure 7B), and TNF-α (Figure 7C) were significantly increased in the pyrogallol (Pyro) group compared to the Control group (*p* < 0.001). Co-treatment with GPE-Ag and pyrogallol (GPE-Ag + Pyro) reduced these levels compared to the Pyro group (*p* < 0.001), but they were still higher than in the Control group. IL-10 (Figure 7D) was significantly increased in the GPE-Ag + Pyro group compared to the Pyro group (*p* < 0.001), showing an anti-inflammatory effect. NF-κB (Figure 8) was also highly increased in the Pyro group (*p* < 0.001) and significantly reduced in the GPE-Ag + Pyro group (*p* < 0.001) as compared to the control group. The GPE-Ag group alone showed no significant changes in any of the measured markers compared to the Control group (*p* > 0.05). Both the GPE-Ag and GPE-Ag + Pyro groups showed clear improvements in all parameters compared to the Pyro group (*p* < 0.001).

### 2.8. Effect of GPE-Ag Nanoparticles on Oxidative Stress Biomarkers in Pyrogallol-Intoxicated Rats

**Figure 9:**
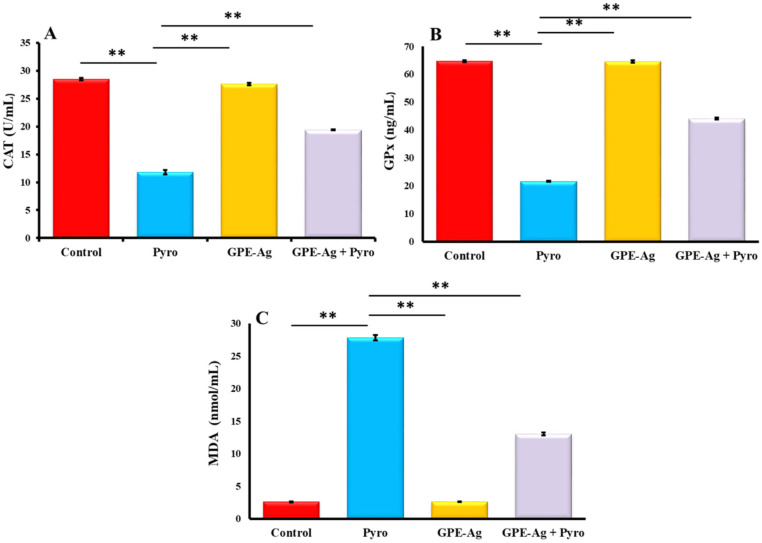
Effect of garlic peel extract-synthesized silver nanoparticles (GPE-Ag) on on hepatic oxidative stress biomarkers; (**A**) CAT, (**B**) GPx, (**C**) MDA in pyrogallol (Pyro)-intoxicated rats. Data expressed as mean values ± SE (*n* = 6/group). ** indicates *p* < 0.001 vs. the Pyro group.

As illustrated in Figure 9A–C, the activities of CAT (Figure 9A) and GPx (Figure 9B) were significantly reduced in the pyrogallol (Pyro) group compared to the control group (*p* < 0.001). In contrast, co-treatment with garlic peel-extract-synthesized silver nanoparticles (GPE-Ag) (GPE-Ag + Pyro group) significantly increased CAT and GPx activities compared to the Pyro group (*p* < 0.001), although these levels did not fully return to normal. Additionally, MDA levels (Figure 9C) were markedly elevated in the Pyro group versus control (*p* < 0.001), while a significant reduction was observed in the GPE-Ag + Pyro group compared to the Pyro group (*p* < 0.001). Despite this improvement, MDA levels remained higher than those in the control group. These findings indicate that GPE-Ag alleviated oxidative stress but did not completely restore redox balance.

### 2.9. Effect of GPE-Ag Nanoparticles on Liver Histopathology in Pyrogallol-Intoxicated Rats

**Figure 10:**
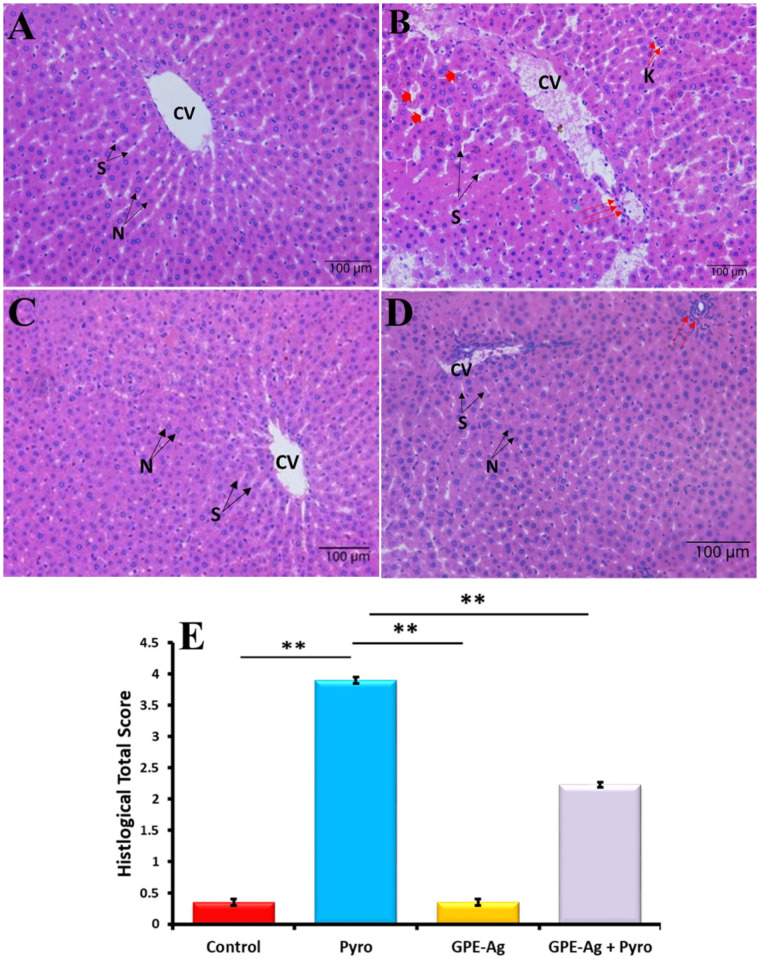
Representative photomicrographs of H&E-stained liver sections from all experimental groups. (**A**) Control group showing normal hepatic architecture, including central vein (CV), hepatic sinusoids (S), and vesicular nuclei (N). (**B**) Pyro-intoxicated group showing a dilated and congested central vein with hemorrhage (CV), necrotic hepatocytes (red arrowhead), mildly dilated sinusoids (S), inflammatory cell infiltration (red arrows), and activated Kupffer cells (K). (**C**) GPE–Ag-treated group displaying nearly normal hepatic structure with intact nuclei (N) and normal sinusoids (S). (**D**) GPE–Ag + Pyro group exhibiting improved hepatic architecture with mostly normal hepatocytes and nuclei (N), slightly dilated central vein (CV), and minimal inflammatory cells (red arrows). (**E**) Histological scores of liver tissue presented as mean ± SE (*n* = 6/group); ** indicates *p* < 0.001 vs. Pyro group. Original magnification: 100×.

Histological examination of hematoxylin and eosin (H&E) stained liver sections revealed distinct morphological changes across the experimental groups. The control group exhibited preserved hepatic architecture with normal hepatocyte morphology, well-defined nuclei, and clearly outlined sinusoids surrounding the central vein, with no signs of necrosis or inflammation (Figure 10A). In contrast, the Pyrogallol (Pyro)-treated group displayed significant hepatic damage, including moderate hepatocellular necrosis, dilated and congested central veins, pyknotic nuclei, widened sinusoids, inflammatory cell infiltration, and Kupffer cell activation, indicating oxidative stress-induced injury (Figure 10B). Treatment with garlic peel extract-synthesized silver nanoparticles (GPE-Ag) alone showed no adverse effects, as liver sections appeared nearly normal, with intact hepatocytes and non-congested sinusoids (Figure 10C). Strikingly, co-treatment with GPE-Ag and Pyro resulted in marked histological improvement, characterized by restored hepatic architecture, minimal inflammation, and only slight dilation of central veins, suggesting a protective effect against Pyro-induced damage (Figure 10D). Semi-quantitative scoring revealed a significant increase in hepatic injury in the pyrogallol-treated group compared with the control (*p* < 0.001), characterized by extensive hepatocellular necrosis, sinusoidal dilatation, and dense inflammatory infiltration. In contrast, treatment with GPE–Ag nanoparticles markedly reduced the injury scores (*p* < 0.001 vs. Pyro-only), reflecting substantial preservation of hepatic architecture. Specifically, the Pyro-only group displayed predominant grades 3–4, indicating severe to very severe hepatic lesions, while the GPE–Ag + pyrogallol co-treated group exhibited mainly grades 1–2, consistent with mild to moderate alterations. These findings confirm the hepatoprotective potential of GPE–Ag nanoparticles against pyrogallol-induced hepatotoxicity (Figure 10E).

## 3. Discussion

This study pioneers the application of silver nanoparticles synthesized from garlic peel extract (GPE–Ag) as a novel, sustainable, and effective hepatoprotective agent in a chemically induced model of liver injury. With liver diseases increasingly linked to oxidative stress and inflammation, current treatments like NAC and silymarin face challenges such as poor bioavailability and side effects [22,23]. Recent studies highlight the growing use of green synthesis from over 65 medicinal plants, emphasizing the value of eco-friendly and biocompatible nanomaterials for future biomedical applications [31]. GPE–Ag offers a promising alternative by enhancing therapeutic delivery while addressing these limitations. Moreover, the use of garlic peel extract as a green synthesis agent supports the development of eco-friendly nanomedicine for liver disease management [66]. The present study is the first to report the synthesis of silver nanoparticles using garlic peel extract (GPE–AgNPs), an underutilized agro-waste by-product, demonstrating a sustainable and eco-friendly nanotechnological approach. The HPLC-based phytochemical profiling confirmed the presence of polyphenolic compounds that served both as reducing and stabilizing agents during nanoparticle formation (Table 1). The reduction of Ag^+^ ions by garlic peel phytochemicals and stabilization through natural capping agents aligns with the principle of green chemistry [67].

Furthermore, the characterization results confirmed the successful synthesis and stability of GPE–Ag nanoparticles. UV–visible spectroscopy revealed a distinct SPR peak at 386 nm, which is in agreement with previous reports on phytogenic silver nanoparticles [68]. In addition, the TEM, DLS, and zeta potential results confirm the formation of capped, moderately stable nanoparticles.

The HPLC profile of the ethanolic garlic peel extract (Table 1) revealed a high abundance of phenolic acids and flavonoids, including ellagic, gallic, and syringic acids, together with kaempferol, quercetin, myricetin, and other related compounds, which are known to act as potent reducing and capping agents during silver nanoparticle synthesis. These phytochemicals, particularly flavonoids and phenolic acids bearing negatively charged hydroxyl and carboxyl groups, interact electrostatically with Ag^+^ ions, facilitating their reduction to Ag^0^ and forming a stabilizing capping layer that enhances colloidal stability while preventing nanoparticle aggregation. [47,58,59,60,62,65,69,70]. Importantly, this work provides the first mechanistic evidence linking the polyphenolic capping of GPE–Ag to their antioxidant and NF-κB–modulated anti-inflammatory effects in a pyrogallol-induced acute hepatotoxicity model, thereby highlighting the potential of garlic peel valorization in green nanomedicine and hepatic protection.

Liver function and histopathological analyses consistently indicated that GPE–Ag mitigated pyrogallol-induced hepatic injury. Pyrogallol administration markedly increased serum AST, ALT, ALP, and GGT levels, reflecting membrane leakage and hepatocellular damage [13,71,72,73]. Treatment with GPE–Ag significantly normalized these enzyme activities, suggesting effective preservation of hepatic structure and function. This protective response aligns with previous findings on antioxidant and anti-inflammatory nanomaterials [74,75]. The observed effect can be attributed to bioactive constituents of garlic peel—particularly flavonoids, phenolic acids, and other related compounds—which, as highlighted in our findings and supported by previous reports, modulate oxidative stress, suppress inflammatory mediators, and promote hepatic recovery [45,49,76,77,78,79,80].

A key finding of this study is the potent antioxidant activity of GPE–Ag, as evidenced by a significant increase in GPx and CAT activities, along with a marked decrease in MDA levels. These changes indicate that GPE–Ag effectively mitigates oxidative stress and preserves cellular redox homeostasis. This antioxidant response is likely driven by the phytochemical constituents involved in nanoparticle synthesis, which not only facilitate silver ion reduction but also enhance endogenous defense mechanisms [81]. Similar antioxidative and cytoprotective effects have been reported for silver nanoparticles synthesized from other botanical sources, including garlic and onion peel extracts [39]. Comparable antioxidant and liver-protective effects have been reported for silver nanoparticles synthesized from plant phenolics such as *Azadirachta indica*, catechol derivatives, and other polyphenol-rich extracts [27,39,40,41,82,83].

Pyrogallol induces oxidative stress in hepatocytes by generating excessive reactive oxygen species (ROS), which in turn activate the NF-κB signaling pathway. This activation promotes the nuclear translocation of NF-κB p65, leading to the upregulation of pro-inflammatory cytokines such as TNF-α, IL-1β, and IL-6. The sustained activation of NF-κB contributes to inflammatory cell infiltration, hepatocellular necrosis, and overall hepatic injury. Thus, pyrogallol serves as a potent model compound for studying ROS-mediated NF-κB–driven inflammatory hepatotoxicity [13,16,84,85,86]. GPE–Ag treatment significantly attenuated oxidative damage while also suppressing key pro-inflammatory cytokines, including IL-1, IL-6, and TNF-α. This was accompanied by downregulation of NF-κB, a master regulator of inflammatory gene expression [87,88]. Interestingly, GPE–Ag also upregulated the expression of IL-10, an anti-inflammatory cytokine known for its immunomodulatory effects [89,90].

Several phytochemicals identified in the garlic peel extract, including quercetin, apigenin, chlorogenic acid, gallic acid, ferulic acid, and caffeic acid, are well known for their ability to enhance antioxidant defenses and protect cellular integrity by activating signaling pathways such as Nrf2/HO-1, AMPK, and SIRT1. Concurrently, these compounds exert anti-inflammatory effects by suppressing NF-κB [91,92,93,94,95,96]. The presence of these bioactive molecules in the garlic peel extract and their synergistic interactions likely contribute to the potent hepatoprotective effects observed with GPE-Ag treatment, reflecting their coordinated role in balancing oxidative stress and inflammatory responses.

In line with these reports, the present work demonstrates that garlic peel–derived polyphenols not only mediate green synthesis of GPE–AgNPs but also amplify their antioxidant and NF-κB–modulated anti-inflammatory responses, providing strong mechanistic evidence for their hepatoprotective efficacy.

NF-κB plays a dual role in hepatic pathology, as its activation not only promotes inflammation but is also involved in tissue repair and apoptosis regulation through IL-10 signaling [97]. The ability of GPE–Ag to influence both pro- and anti-inflammatory branches of the NF-κB pathway suggests a balanced immunomodulatory effect, which may be particularly advantageous in managing chronic liver inflammation [98]. Recent mechanistic evidence supports that phenolic compounds such as ellagic acid, rutin, myricetin, syringic acid, and catechin can decrease hepatocellular oxidative injury by enhancing the phosphorylation of AMPK and upregulating Nrf2/HO-1 expression. This pathway works together to reduce the release of IL-6, TNF-α, and IL-1β [2,91,94,99,100]. This mechanistic overlap supports the finding role of GPE–Ag in regulating redox and cytokine activity at the molecular level.

Natural antioxidants are well recognized for enhancing cellular antioxidant defenses and inhibiting inflammation-driven liver damage [27]. These findings are supported by earlier studies demonstrating that plant-based compounds and green-synthesized silver nanoparticles can effectively block pathways such as Akt (protein kinase B) and NF-κB, thereby reducing cytokine release and promoting anti-inflammatory responses [101,102,103,104,105,106].

Furthermore, additional molecular investigations are necessary to elucidate its precise intracellular mechanisms. Addressing these gaps is essential for advancing GPE–Ag toward clinical application. When compared with conventional hepatoprotective agents, GPE–Ag demonstrates several advantageous features. For instance, silymarin nanoformulations have been shown to enhance the bioavailability and therapeutic efficacy of silymarin in treating hepatotoxicity and liver fibrosis, primarily through potent antioxidant, anti-inflammatory, and membrane-stabilizing actions that preserve hepatocellular integrity [92,107,108,109]. Whereas NAC is mainly known for its detoxifying action [110,111,112]. In contrast, GPE–Ag integrates a broader spectrum of protective properties—including high concentrations of polyphenols –induced antioxidant, anti-inflammatory, detoxifying, and membrane-stabilizing effects—highlighting its potential as a more versatile and comprehensive therapeutic agent. These comparative features are outlined in Table 2. A comprehensive analysis of the present findings suggests that GPE–Ag exerts its hepatoprotective effects through a multifaceted mechanism. These include (i) direct neutralization of reactive oxygen species (ROS), (ii) enhancement of endogenous antioxidant enzyme activity, and (iii) inhibition of the NF-κB signaling cascade, resulting in the downregulation of pro-inflammatory cytokines.

In summary, the concurrent reduction in reactive oxygen species (ROS) and suppression of inflammatory mediators highlight the multifunctional therapeutic potential of GPE–Ag nanoparticles in hepatoprotection. The eco-friendly synthesized GPE–Ag nanoparticles, rich in polyphenolic constituents from garlic peel extract, exhibited strong antioxidant defense, inhibited NF-κB–mediated inflammation, and preserved hepatic architecture. Despite these promising outcomes, certain limitations should be acknowledged. While the findings confirm the preclinical efficacy of GPE–Ag nanoparticles, the study was restricted to an acute rat model and lacks long-term in vivo data on chronic toxicity, biodistribution, and pharmacokinetics. Additionally, NF-κB levels were quantified using ELISA without protein-level validation (e.g., Western blot). The absence of FTIR and Raman spectroscopy limits direct confirmation of surface functionalization, and XRD or SAED analyses were not performed to verify crystallinity due to equipment unavailability. These aspects will be addressed in future studies to strengthen the structural, mechanistic, and biosafety understanding of GPE–Ag nanoparticles.

## 4. Materials and Methods

### 4.1. Plants

Garlic bulbs were sourced from a local market in Al-Ahsa, Saudi Arabia. The dry inner peels were carefully separated by hand, washed thoroughly with sterile distilled water, and left to air dry for 60 min until a stable weight was achieved, thereby preserving thermolabile bioactive compounds, as described by [115]. The dried peels were then finely ground using an 80-mesh sieve and stored for subsequent ethanolic extraction.

### 4.2. Preparation of Garlic Peel Ethanolic Extract (GPE)

The powdered garlic peels were subjected to maceration following standard pharmacognostic procedures as described by [116]. Specifically, 300 g of the dried garlic peel powder was soaked in of (aqueous, 95%) ethanol (1:4 *w*/*v*) was purchased from Sigma-Aldrich (St. Louis, MO, USA) and kept at room temperature for 72 h with intermittent shaking to facilitate extraction of phytochemicals. The mixture was then filtered through sterile muslin cloth followed by Whatman No. 1 filter paper (24 cm). The combined filtrate was concentrated using a Büchi Rotavapor^®^ R-300 (BÜCHI Labortechnik AG, Flawil, Switzerland) under reduced pressure at 40 °C to remove excess ethanol. The resulting semisolid extract was stored in an amber-colored sterile container at −20 °C prior to use in silver nanoparticle synthesis.

### 4.3. HPLC Analysis of Polyphenols in Garlic Peel Extract (GPE)

Polyphenolic compounds in the ethanolic extract of Lepidium sativum seeds were analyzed using HPLC following a modified version of [117]. Separation was performed on an Eclipse XDB-C18 column (125 × 4.6 mm, 5 µm) with a C18 guard column (Phenomenex, Torrance, CA, USA), using an Agilent 1100 series HPLC system (Agilent Technologies, Waldbronn, Germany) equipped with an autosampler and diode-array detector. The mobile phase consisted of solvent A (2% acetic acid in water, *v*/*v*) and solvent B (acetonitrile), applied in a gradient program: 8–12% B over 10 min, 12–35% B over 50 min, followed by re-equilibration to the initial composition within 5 min, for a total run time of 60 min. The flow rate was 1.0 mL/min, and the injection volume was 20 µL. Detection was performed at 280 nm. All samples were filtered through 0.45 µm syringe filters prior to injection. Peaks were identified by comparing retention times and UV spectra with authentic standards, and quantification was performed using the external standard calibration method.

### 4.4. Synthesis of Garlic Peel Extract-Silver Nanoparticles (GPE–Ag)

0.1 M silver nitrate (AgNO_3_ was obtained from Sigma-Aldrich (St. Louis, MO, USA) stock solution was prepared by dissolving 1.7 g of AgNO_3_ in 100 mL of distilled water and stored in the dark to prevent photodecomposition. 1 mL of this stock solution (equivalent to 5 mM AgNO_3_), For each synthesis, 5 g of standardized garlic peel extract was mixed with 50 mL stock solution silver nitrate at room temperature (25 °C). The reaction mixture was magnetically stirred for 10 min, and the pH was adjusted to 8–10 using 0.1 M NaOH. The reduction of Ag^+^ to Ag^0^ was evidenced by a color change from pale yellow to deep brown and further confirmed by the appearance of a characteristic SPR peak, indicative of silver nanoparticle (AgNP) formation. All synthesis parameters—including extract-to-silver ratio, temperature, pH, and stirring time—were optimized and maintained constant across replicates to ensure consistent nanoparticle yield and reproducibility. The nanoparticles were harvested by using a Hermle Z326K centrifuge (Hermle Labortechnik GmbH, Wehingen, Germany) at 2400× *g* for 30 min at 10 °C, followed by repeated washing with methanol and distilled water to remove unbound phytochemicals and residual ions. The purified AgNPs were then dried in a hot air oven (Memmert UN30, Memmert GmbH + Co. KG, Schwabach, Germany) at 25 °C. The dried nanoparticles were stored and subsequently used for further characterization and analysis [118].

### 4.5. Characterization of Silver Nanoparticles

The successful biosynthesis of silver nanoparticles (AgNPs) was initially confirmed by ultraviolet–visible (UV–Vis) spectroscopy using a Genway UV-1600 spectrophotometer (Genway, San Diego, CA, USA), with spectra recorded to identify the characteristic SPR peak. The morphology and size of the synthesized GPE–Ag were further characterized by High-Resolution Transmission Electron Microscopy (HR-TEM) that offered detailed insights into nanoparticle size, shape, and crystalline structure using instruments from JEOL Ltd. (Tokyo, Japan). TEM analysis (performed using ImageJ software version 1.53k) was used to determine the mean core size, morphology, and particle dispersion of the GPE–Ag nanoparticles. Scanning Electron Microscopy (SEM; JEOL Ltd., Tokyo, Japan) was employed to examine surface topology and aggregation patterns. DLS analysis was conducted using a Zetasizer Nano ZS (Malvern Panalytical Ltd., Malvern, UK) to determine the hydrodynamic diameter and PDI of the nanoparticles in aqueous suspension. For DLS measurements, 30 μL of the GPE–AgNP suspension was diluted in 1 mL of deionized water, and each sample was analyzed in triplicate at room temperature (22 °C). Zeta potential was measured using a ZetaPALS zeta potential analyzer (Brookhaven Instruments Corporation, Holtsville, NY, USA) based on the DLS principle to evaluate the surface charge and colloidal stability of the synthesized nanoparticles. Elemental composition was analyzed using EDAX integrated with a field emission scanning electron microscope (Area1-SEM). The data were acquired and processed using APEX™ software (version 3.0; EDAX Inc., Mahwah, NJ, USA) to determine the elemental distribution and weight percentages to confirm the presence of elemental silver and assess sample purity.

### 4.6. Animals

Male albino rats (aged approximately 6 weeks, weighing 150–170 g) were procured from the animal care facility at King Faisal University, Saudi Arabia. The animals were housed under standard laboratory conditions, including a controlled temperature of 24 ± 1 °C and a 12-h light/dark photoperiod. They had unrestricted access to a standard rodent chow and clean drinking water throughout the study. Prior to the initiation of the experimental procedures, all rats underwent a 2-week acclimatization period to adapt to the housing environment. All animal handling and experimental protocols were conducted in compliance with ethical guidelines and were approved by the Research Ethics Committee of King Faisal University (Approval No. KFU-REC-2025-FEB-ETHICS3506).

### 4.7. Experimental Design

The rats were randomly assigned into four groups (*n* = 6 per group), as illustrated schematically in Table 3, which summarizes the treatment design, dosing regimen, and routes of administration applied during the 28-day experimental period.

- Group 1 (Control): received oral saline (0.9%, 5 mL/kg) daily and a single intraperitoneal (i.p.) injection of saline (0.9%, 1 mL/kg) on day 14.
- Group 2 (Pyro): received oral saline (0.9%, 5 mL/kg) daily and a single i.p. injection of pyrogallol (100 mg/kg in saline, 1 mL/kg) on day 14 to induce hepatotoxicity.
- Group 3 (GPE–Ag): received GPE-synthesized silver nanoparticles (GPE–Ag) orally at 50 mg/kg/day (suspended in saline, 0.9%, 5 mL/kg) for 28 days. On day 14, they received a single i.p. injection of saline (1 mL/kg) and continued GPE–Ag treatment for an additional 14 days.
- Group 4 (Pyro + GPE–Ag): received GPE–Ag orally at 50 mg/kg/day as in Group 3; on day 14, hepatotoxicity was induced by a single i.p. injection of pyrogallol (100 mg/kg in saline, 1 mL/kg), and GPE–Ag treatment continued for a further 14 days (total treatment period 28 days).

The dose and schedule of pyrogallol for inducing hepatotoxicity in rats were adopted from previous studies [12], while the GPE–Ag dose (50 mg/kg/day for 28 days) was selected based on earlier reports demonstrating safe and effective use of herbal-mediated silver nanoparticles [119,120].

### 4.8. Sample Collection and Tissue Processing

At the end of the experimental period, all animals were euthanized by inhalation of ether overdose, six hours following pyrogallol administration. Blood and liver samples were promptly collected for biochemical, histological, and immunological analyses. Whole blood was allowed to clot at room temperature for 15–30 min and then centrifuged at 1000× *g* for 10 min using a benchtop centrifuge (Hettich Zentrifugen, Hamburg, Germany) to separate the serum, which was stored at −20 °C for subsequent liver function analysis. The liver was immediately excised and divided into two portions: one was snap-frozen in liquid nitrogen and stored at −80 °C for biochemical and enzyme-linked immunosorbent assay (ELISA) based assessments, while the other was fixed in 10% neutral buffered formalin (Sigma-Aldrich, St. Louis, MO, USA) for histopathological evaluation. For biochemical assays, a defined weight of liver tissue was homogenized in ice-cold 0.1 M phosphate buffer (pH 7.0–7.2; Thermo Fisher Scientific, Waltham, MA, USA) using a Teflon-glass homogenizer (IKA-Werke GmbH & Co. KG, Staufen, Germany). The homogenates were centrifuged at 2800× *g* for 30 min at 4 °C using a refrigerated centrifuge (Eppendorf 5810 R, Hamburg, Germany), and the resulting supernatants were stored at −80 °C until analysis.

### 4.9. Determination of Serum Liver Enzymes via Colorimetric Assay

The enzymatic activities of key liver function biomarkers—alanine aminotransferase (ALT/GPT), aspartate aminotransferase (AST/GOT), alkaline phosphatase (ALP), and gamma-glutamyl transferase (GGT)—were quantified using specific colorimetric assay kits. The assays were conducted following the manufacturer’s protocols using kits with catalog numbers EEA001 (ALT), EEA003 (AST), EEA002 (ALP), and EEA029 (GGT) from Invitrogen (Thermo Fisher Scientific, Carlsbad, CA, USA). Enzyme activities were measured using a spectrophotometric microplate reader (Molecular Devices, San Jose, CA, USA) at their designated wavelengths, and results were expressed in units per liter (U/L). Liver function assay sensitivities were as follows: GOT (1.1 IU/L), GPT (0.75 IU/L), ALP (0.13 IU/L), and GGT (0.88 IU/L), with intra-assay < 10% and inter-assay < 12%.

### 4.10. Assessment of Hepatic Immunological and Molecular Markers Using ELISA

The concentrations of key pro- and anti-inflammatory cytokines—including TNF-α, IL-1, IL-6, IL-10, and the transcription factor NF-κB—were quantitatively assessed in liver tissue homogenates using rat-specific ELISA kits. The assays were performed according to the manufacturer’s protocols, using the following catalog numbers: TNF-α (Cat. No. MBS267737), IL-1(Cat. No. MBS264984), IL-6 (Cat. No. MBS495243), IL-10 (Cat. No. MBS2503826), and NF-κB (Cat. No. MBS453975), all obtained from MyBioSource Inc. (San Diego, CA, USA). Standards and samples were run in duplicate, and absorbance was measured at 450 nm using a microplate reader (BioTek Instruments, Winooski, VT, USA). The assay sensitivities were as follows: IL-1, IL-6, and TNF-α (5 pg/mL), IL-10 (18.75 pg/mL), and NF-κB (0.119 ng/mL). Intra-assay and inter-assay precision values were ≤8% and ≤12%, respectively, except for IL-10, which showed <7% and <6%, respectively.

### 4.11. Assessment of Hepatic Redox Balance

The activities of key antioxidant enzymes (CAT) and GPx were measured in liver tissue homogenates using rat-specific ELISA kits according to the manufacturer’s protocols. The kits used were CAT (Cat. No. MBS006963), and GPx (Cat. No. MBS744364), all obtained from MyBioSource Inc. (San Diego, CA, USA). The assays exhibited sensitivities of 1.0 U/mL for CAT and 0.1 ng/mL for GPx, with intra- and inter-assay precision values of <15% and <12%, respectively. Lipid peroxidation was evaluated by determining MDA levels using a colorimetric thiobarbituric acid reactive substances (TBARS) assay. In this method, MDA reacts with thiobarbituric acid (TBA) under acidic conditions at 95 °C, forming a pink chromogen that is measured spectrophotometrically at 534 nm [121].

### 4.12. Histological Assessment of Liver Architecture

Liver tissue samples were fixed in 10% neutral buffered formalin at 28 °C for 24 h, followed by standard dehydration through graded alcohols and embedding in paraffin wax. Tissue sections approximately 4 µm thick were prepared using a rotary microtome (Leica RM2235, Leica Biosystems, Wetzlar, Germany) and stained with H&E to evaluate histological alterations. The stained sections were examined under a light microscope (Nikon Corporation, Tokyo, Japan). Histopathological changes were assessed using a semi-quantitative scoring system ranging from 0 to 4, based on the severity of hepatocellular necrosis and inflammatory cell infiltration, as described by [122]. All reagents were obtained from Sigma-Aldrich (St. Louis, MO, USA). Three liver sections from each experimental group were examined under a light microscope (×400). For each section, five randomly selected non-overlapping fields were evaluated to ensure representative sampling. Semi-quantitative histological scoring was performed to assess the degree of liver injury based on hepatocellular necrosis, sinusoidal dilatation, inflammatory cell infiltration, and Kupffer cell activation, using a standardized five-point scale (0–4), where 0 = none, 1 = mild, 2 = moderate, 3 = severe, and 4 = very severe [122]. Scoring was conducted independently by two experienced histopathologists who were blinded to the treatment groups. To ensure unbiased evaluation, all samples were coded prior to examination, preventing any identification of group assignments.

### 4.13. Statistical Analysis

Statistical analyses were performed using Statistical Package for the Social Sciences (SPSS) software, version 24.0 (IBM Corp., Armonk, NY, USA). Categorical data were expressed as frequencies and percentages, while normally distributed continuous data were presented as mean ± standard error (SE). One-way ANOVA was used to compare multiple groups, followed by the Least Significant Difference (LSD) post hoc test for pairwise comparisons. Statistical significance was set at *p* < 0.05 (*) and at *p* < 0.001 (**). Values of *p* > 0.05 were considered not significant.

## 5. Conclusions

GPE–Ag nanoparticles synthesized from garlic peel extract exhibited hepatoprotective effects against pyrogallol-induced liver injury, primarily through antioxidant activation, NF-κB–mediated inflammation suppression, and preservation of hepatic architecture. These results position GPE–Ag as a promising eco-friendly nanoplatform for preventing and managing chemical- and drug-induced hepatotoxicity. While the study confirms efficacy in an acute model, further preclinical and clinical studies are needed to assess long-term safety, biodistribution, and pharmacokinetics to support its translational potential.

## Figures and Tables

**Table 1:** HPLC profile of Polyphenolic compounds in garlic peel extract (GPE).

Compound	R.T.	Class/Subclass	Conc. (µg/gm)	Caps/Stabilizes Ag-NPs (Ag^+^ → Ag^0^)
7-OH Flavone	2.0	Flavonoid/7-hydroxyflavone	4.88	[55]
Chlorogenic acid	3.0	Phenolic/phenolic acid ester	6.20	[56]
Apigenin	4.0	Flavonoid/trihydroxyflavone	4.63	[57]
Ellagic acid	5.0	Phenolic/polyphenolic lactone	18.78	[58]
Gallic acid	6.0	Phenolic/hydroxybenzoic acid	15.20	[59]
Quercetin	7.0	Flavonoid/pentahydroxyl flavonol	14.77	[59]
Syringic acid	8.0	Phenolic/hydroxybenzoic acid	13.89	[60]
Rutin	10.0	Flavonoid/flavonol glycoside	4.25	[61]
Myricetin	11.0	Flavonoid/hexahydroxyflavone	13.76	[62]
Ferulic acid	13.0	Phenolic/hydroxycinnamic acid	7.94	[63]
Cinnamic acid	14.0	Phenolic/cinnamic acid derivative	2.04	[60]
Benzoic acid	15.0	Phenolic-related/benzoic acid derivative	7.64	[60]
Catechin	16.0	Flavonoid/3-flavanol	1.85	[64]
Kaempferol	17.0	Flavonoid (flavonol)	15.67	[65]
Salicylic acid	18.0	Phenolic (benzoic acid derivative)	3.08	[60]

R.T. = Retention time; Conc.= Concentartion.

**Table 2:** Comparative Hepatoprotective Profiles of Clinical Standards and the Novel GPE–Ag Nanoplatform.

Category	Agent	Liver Function	Mechanisms	Adverse/Toxic Effects	Novelty	References
Green Nanomedicine	GPE–Ag	ALT (35.7%) AST (45.7%) ALP (23.4%)	↓ NF-κB (32.4%)	No systemic toxicity at 50 mg/kg; safe below reported LD_50_ (>2000 mg/kg)	Linking garlic peel–derived polyphenolic capping to antioxidant and NF-κB–mediated hepatoprotection; promotes sustainable agro-waste–based nanomedicine	Present study
			↓ IL-1 (28.9%)			
			↓TNF-α (44.9%)			
			↑ IL-10 (52.56%)			
			↑ CAT (64.4%)			
			↓ MDA (113%)			
Synthetic Drug	N-Acetylcysteine (NAC)	↓ ALT, ↓ AST	↑ GSH, ↓ROS, ↓proinflammatory cytokine	Nausea, vomiting, possible hepatotoxicity at >200 mg/kg	Acts primarily via glutathione synthesis; lacks polyphenolic or cytokine-targeted effects	[23,110,112]
Natural Phytotherapeutic	Silymarin/nano	↓ ALT, ↓ AST	↓ lipid peroxidation, ↓proinflammatory cytokine	Mild gastrointestinal discomfort	Phytocomplex with known antioxidant activity; no NF-κB pathway assessment or green nanoplatform integration	[22,109,113,114]

↑ indicates an increase; ↓ indicates a decrease.

**Table 3:** Experimental grouping and treatment schedule.

Group	Designation	Oral Administration (Days 1–28)	Intraperitoneal (i.p.) Injection (Day 14)
Control	Normal saline control	Saline (0.9%, 5 mL/kg/day)	Saline (0.9%, 1 mL/kg)
Pyro	Hepatotoxicity model	Saline (0.9%, 5 mL/kg/day)	Pyrogallol (100 mg/kg in saline)
GPE–Ag	Nanoparticles only	GPE–Ag nanoparticles (50 mg/kg/day)	Saline (0.9%, 1 mL/kg)
Pyro + GPE–Ag	Hepatotoxicity + treatment	GPE–Ag nanoparticles (50 mg/kg/day)	Pyrogallol (100 mg/kg in saline)

## Data Availability

The original contributions presented in this study are included in the article. Further inquiries can be directed to the corresponding authors.
